# Crystal structure of 4-bromo-*N*-[(3,6-di-*tert*-butyl-9*H*-carbazol-1-yl)methyl­idene]aniline

**DOI:** 10.1107/S2056989019012374

**Published:** 2019-09-10

**Authors:** Koji Kubono, Taisuke Matsumoto, Masatsugu Taneda

**Affiliations:** a Division of Natural Sciences, Osaka Kyoiku University, Kashiwara, Osaka 582-8582, Japan; b Institute for Materials Chemistry and Engineering, Kyushu University, Kasuga, Fukuoka 816-8580, Japan; c Department of Science Education, Faculty of Education, Osaka Kyoiku University, Kashiwara, Osaka 582-8582, Japan

**Keywords:** crystal structure, carbazole, Schiff base, intra­molecular hydrogen bond, C—H⋯π inter­action

## Abstract

In the title compound, C_27_H_29_BrN_2_, the carbazole ring system is essentially planar, with an r.m.s. deviation of 0.0781 (16) Å. An intra­molecular N—H⋯N hydrogen bond forms an *S*(6) ring motif. One of the *tert*-butyl substituents shows rotational disorder over two sites with occupancies of 0.592 (3) and 0.408 (3). In the crystal, two mol­ecules are associated into an inversion dimer through a pair of C—H⋯π inter­actions. The dimers are further linked by another pair of C—H⋯π inter­actions, forming a ribbon along the *c*-axis direction. A C—H⋯π inter­action involving the minor disordered component and the carbazole ring system links the ribbons, generating a network sheet parallel to (100).

## Abstract 2

In the title compound, C_27_H_29_BrN_2_, an intra­molecular N—H⋯N hydrogen bond forms an *S*(6) ring motif. In the crystal, two mol­ecules are associated into an inversion dimer *via* a pair of C—H⋯π inter­actions. The dimers are linked by another pair of C—H⋯π inter­actions, forming a ribbon along the *c-*axis direction.

## Chemical context   

Carbazole derivatives have been widely applied in various fields such as pharmaceuticals (Obora, 2018 ▸), electroluminescent materials (Krucaite & Grigalevicius, 2019 ▸; Taneda, *et al.*, 2015 ▸) and dyes (Zhao *et al.*, 2019 ▸). As a result of the high acidity of the N—H bond, 9*H*-carbazoles have also attracted much attention as hydrogen donors in hydrogen-bonding systems (Rubio *et al.*, 2015 ▸; Wiosna-Sałyga *et al.*, 2006 ▸). Substitution of the 1 position of 9*H*-carbazole with a hydrogen acceptor can afford an intra­molecular hydrogen-bonding system in the mol­ecules. In this work, a Schiff base including carbazole, *N*-(3,6-di-*tert*-butyl-9*H*-calbazol-1-yl­methyl­idene)-4-bromo­aniline, is newly synthesized. 3,6-Di-*tert*-butyl-9*H*-carbazole is useful in order to substitute the 1-position of the 9*H*-carbazole moiety because the substitution reaction would only occur at its 1- and 8-positions. Thus, the title compound has two *tert*-butyl groups on the carbazole moiety. The title compound is a suitable model to investigate an intra­molecular hydrogen bond between the heteroaromatic N—H and the N atom of the imino group. We report herein on its mol­ecular and crystal structures.
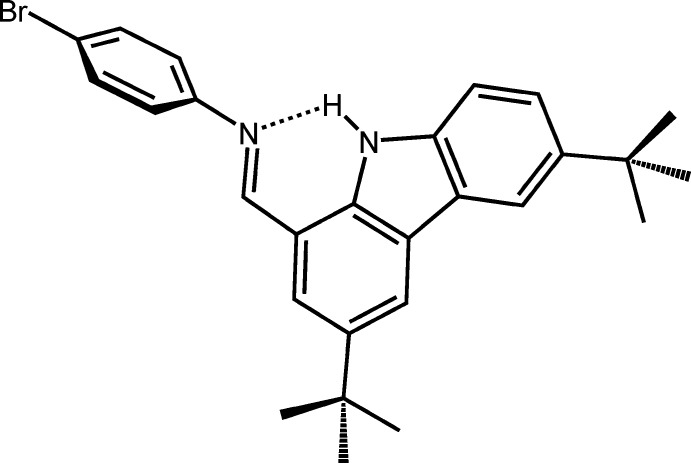


## Structural commentary   

**Figure 1:**
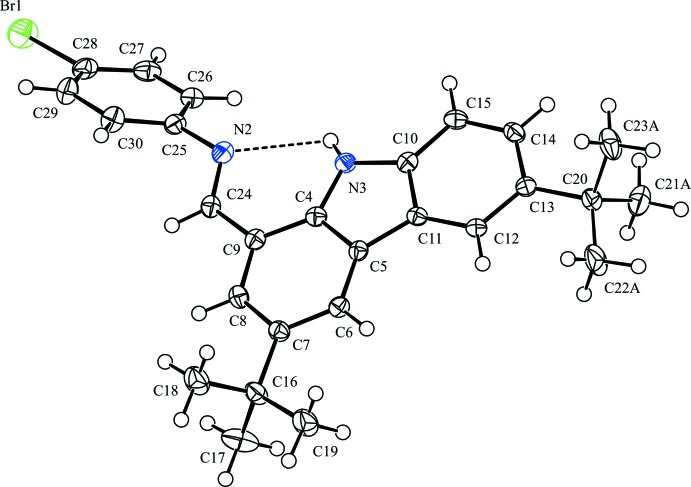
The mol­ecular structure of the title compound, with atom labelling. Only the major disordered component is shown. Displacement ellipsoids are drawn at the 50% probability level. H atoms are represented by spheres of arbitrary radius. The intra­molecular N—H⋯N hydrogen bond is shown as a dashed line.

The mol­ecular structure of the title compound is shown in Fig. 1 ▸. The mol­ecule adopts an *E* configuration with respect to the C=N double bond. The carbazole ring is almost planar with a maximum deviation of 0.0781 (16) Å at atom C8. There is an intra­molecular N—H⋯N hydrogen bond involving the amino group (N3—H3) in the carbazole ring and an imine N atom (N2), generating an *S*(6) ring motif (Table 1 ▸). The dihedral angle between the mean planes of the carbazole ring system and the benzene C25–C30 ring is 42.72 (7)°. The bond lengths and angles of the title compound are normal and agree with those values in other carbazole imine compounds (Gibson *et al.*, 2003 ▸; Nolla-Saltiel *et al.*, 2018 ▸). One of the *tert*-butyl substituents shows rotational disorder around the C13—C20 bond axis over two sites with occupancies of 0.592 (3) and 0.408 (3).

## Supra­molecular features   

**Figure 2:**
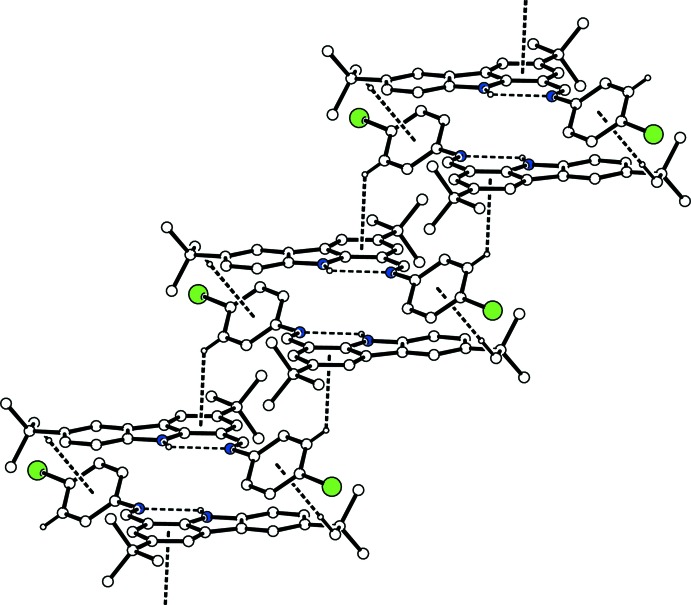
A packing diagram of the title compound, showing the ribbon structure. The N—H⋯N hydrogen bonds and the C—H⋯π inter­actions are shown as dashed lines. H atoms not involved in the inter­actions and the minor disorder component have been omitted for clarity.

**Figure 3:**
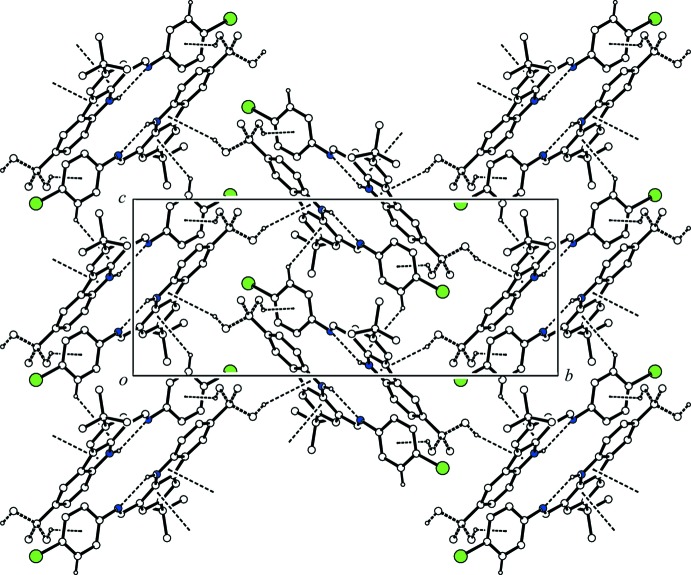
A packing diagram of the title compound viewed along the *a* axis, showing a sheet structure. The minor disorder component is shown with bold dashed lines. The N—H⋯N hydrogen bonds and the C—H⋯π inter­actions are shown as dashed lines. H atoms not involved in the inter­actions and the major disorder component have been omitted for clarity.

In the crystal, two mol­ecules are associated through a pair of C—H⋯π inter­actions (C22*A*—H22*C*⋯*Cg*1^i^ in the major disorder component or C21*B*—H21*E*⋯*Cg*1^i^ in the minor disorder component; *Cg*1 is the centroid of the C25–C30 ring; symmetry code as in Table 1 ▸), forming a centrosymmetric dimer. The dimers are linked by another pair of C—H⋯π inter­actions (C29—H29⋯*Cg*2^ii^; *Cg*2 is the centroid of the C4–C9 ring; symmetry code as in Table 1 ▸), forming a ribbon along the *c-*axis direction (Fig. 2 ▸). These ribbons are linked *via* a C—H⋯π inter­action involving the minor disorder component (C22*B*—H22*D*⋯*Cg*3^iii^; *Cg*3 is the centroid of the N3/C4/C5/C11/C10 ring; symmetry code as in Table 1 ▸) into a network sheet parallel to (100) (Fig. 3 ▸).

## Database survey   

A search of the Cambridge Structural Database (CSD, Version 5.40; February 2019; Groom *et al.*, 2016 ▸) gave 56 and 5 hits, respectively, for the 3,6-di-*tert*-butyl-9*H*-carbazole and 9*H*-carbazol-1-yl­methyl­idene fragments. Of these structures, the compounds that resemble the title compound are (3,6-di-tert-butyl-9*H*-carbazole-1,8-di­yl)bis­[*N*-(naphthalen-1-yl)methanimine] (Nolla-Saltiel *et al.*, 2018 ▸) and 1,8-bis­[(2,4,6-tri­methyl­phen­yl)imino­meth­yl]-3,6-dimethyl-9*H*-carbazole (Gibson *et al.*, 2003 ▸).

## Synthesis and crystallization   

3,6-Di-*tert*-butyl-9*H*-carbazole-1-carbaldehyde (154 mg, 0.50 mmol) and 4-bromo­aniline (86 mg, 0.50 mmol) were treated in xylene (10 ml) at 423 K under inert gas overnight, followed by evaporation. The recrystallization of the residue from a solvent mixture of acetone and methanol (1:1, *v*:*v*) afforded single crystals of the title compound suitable for X-ray structure analysis (97 mg, 0.21 mmol; yield 42%). ^1^H NMR (CDCl_3_, 400 MHz) *δ* = 1.47 [*s*, 9H, C(CH_3_)_3_], 1.49 [*s*, 9H, C(CH_3_)_3_], 7.22 (*td*, 2H, *J_ortho_* = 8.6 Hz, *J_meta_* = 2.4 Hz, ArH), 7.47–7.58 (*m*, 4H, ArH), 7.67 (*d*, 1H, *J_meta_* = 1.8 Hz, ArH), 8.13 (*d*, 1H, *J_meta_* = 1.8 Hz, ArH), 8.26 (*d*, 1H, *J_meta_* = 1.7 Hz, ArH), 8.72 (*s*, 1H, CH=N), 10.55 (*b*, 1H, NH). HR–MS (*m*/*z*): calculated for [C_27_H_30_BrN_2_]^+^, *m*/*z* = 461.1587; found, 461.1627.

## Refinement   

Crystal data, data collection and structure refinement details are summarized in Table 2 ▸. The H atom attached to atom N3 was located in a difference-Fourier map and freely refined. The C-bound H atoms were positioned geometrically (C—H = 0.93–0.96 Å) and refined using a riding model with *U*_iso_(H) = 1.2*U*_eq_(C). Orientational disorder of the *tert*-butyl substituent (C20–C23) around the C13—C20 bond axis is observed and the occupancies refined to 0.592 (3) and 0.408 (3).

## Supplementary Material

Crystal structure: contains datablock(s) global, I. DOI: 10.1107/S2056989019012374/is5522sup1.cif

Structure factors: contains datablock(s) I. DOI: 10.1107/S2056989019012374/is5522Isup2.hkl

Supporting information file. DOI: 10.1107/S2056989019012374/is5522Isup3.cml

CCDC reference: 1951647

Additional supporting information:  crystallographic information; 3D view; checkCIF report

## Figures and Tables

**Table 1:** Hydrogen-bond geometry (Å, °) *Cg*1, *Cg*2 and *Cg*3 are the centroids of the C25–C30, C4–C9 and N3/C4/C5/C11/C10 rings, respectively.

*D*—H⋯*A*	*D*—H	H⋯*A*	*D*⋯*A*	*D*—H⋯*A*
N3—H3⋯N2	0.76 (2)	2.39 (2)	2.862 (2)	121.3 (16)
C22*A*—H22*C*⋯*Cg*1^i^	0.96	2.92	3.878 (4)	177
C29—H29⋯*Cg*2^ii^	0.93	2.95	3.613 (2)	129
C21*B*—H21*E*⋯*Cg*1^i^	0.96	2.62	3.391 (5)	138
C22*B*—H22*D*⋯*Cg*3^iii^	0.96	2.92	3.839 (5)	159

Symmetry codes: (i) 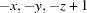; (ii) 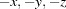; (iii) 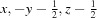.

**Table 2:** Experimental details

Crystal data	
Chemical formula	C_27_H_29_BrN_2_
*M*_r_	461.42
Crystal system, space group	Monoclinic, *P*2_1_/*c*
Temperature (K)	123
*a*, *b*, *c* (Å)	9.9949 (5), 23.546 (1), 10.2919 (6)
β (°)	108.334 (6)
*V* (Å^3^)	2299.2 (2)
*Z*	4
Radiation type	Mo *K*α
μ (mm^−1^)	1.80
Crystal size (mm)	0.40 × 0.30 × 0.20

Data collection	
Diffractometer	Rigaku AFC HyPix-6000
Absorption correction	Multi-scan (*CrysAlis PRO*; Rigaku OD, 2018 ▸)
*T*_min_, *T*_max_	0.610, 0.696
No. of measured, independent and observed [*F* ^2^ > 2.0σ(*F* ^2^)] reflections	19373, 5268, 4580
*R*_int_	0.025
(sin θ/λ)_max_ (Å^−1^)	0.649

Refinement	
*R*[*F* ^2^ > 2σ(*F* ^2^)], *wR*(*F* ^2^), *S*	0.034, 0.076, 1.03
No. of reflections	5268
No. of parameters	312
H-atom treatment	H atoms treated by a mixture of independent and constrained refinement
Δρ_max_, Δρ_min_ (e Å^−3^)	0.56, −0.54

Computer programs: *CrysAlis PRO* (Rigaku OD, 2018 ▸), *SIR92* (Altomare *et al.*, 1993 ▸), *SHELXL2014* (Sheldrick, 2015 ▸), *PLATON* (Spek, 2009 ▸) and *CrystalStructure* (Rigaku, 2016 ▸).

## supplementary crystallographic information

### Crystal data

**Table:**

C_27_H_29_BrN_2_	*F*(000) = 960.00
*M**_r_* = 461.42	*D*_x_ = 1.333 Mg m^−^^3^
Monoclinic, *P*2_1_/*c*	Mo *K*α radiation, λ = 0.71075 Å
*a* = 9.9949 (5) Å	Cell parameters from 8890 reflections
*b* = 23.546 (1) Å	θ = 2.3–30.3°
*c* = 10.2919 (6) Å	µ = 1.80 mm^−^^1^
β = 108.334 (6)°	*T* = 123 K
*V* = 2299.2 (2) Å^3^	Prism, yellow
*Z* = 4	0.40 × 0.30 × 0.20 mm

### Data collection

**Table:**

Rigaku AFC HyPix-6000 diffractometer	5268 independent reflections
Radiation source: rotating anode X-ray generator, FR-E+	4580 reflections with *F*^2^ > 2.0σ(*F*^2^)
Multi-layer mirror optics monochromator	*R*_int_ = 0.025
Detector resolution: 10.0 pixels mm^-1^	θ_max_ = 27.5°, θ_min_ = 2.3°
ω scans	*h* = −10→12
Absorption correction: multi-scan (CrysAlis PRO; Rigaku OD, 2018)	*k* = −26→30
*T*_min_ = 0.610, *T*_max_ = 0.696	*l* = −13→13
19373 measured reflections	

### Refinement

**Table:**

Refinement on *F*^2^	Secondary atom site location: difference Fourier map
*R*[*F*^2^ > 2σ(*F*^2^)] = 0.034	Hydrogen site location: inferred from neighbouring sites
*wR*(*F*^2^) = 0.076	H atoms treated by a mixture of independent and constrained refinement
*S* = 1.03	*w* = 1/[σ^2^(*F*_o_^2^) + (0.0306*P*)^2^ + 1.3852*P*] where *P* = (*F*_o_^2^ + 2*F*_c_^2^)/3
5268 reflections	(Δ/σ)_max_ < 0.001
312 parameters	Δρ_max_ = 0.56 e Å^−^^3^
0 restraints	Δρ_min_ = −0.54 e Å^−^^3^
Primary atom site location: structure-invariant direct methods	

### Special details

**Table:**

Geometry. All esds (except the esd in the dihedral angle between two l.s. planes) are estimated using the full covariance matrix. The cell esds are taken into account individually in the estimation of esds in distances, angles and torsion angles; correlations between esds in cell parameters are only used when they are defined by crystal symmetry. An approximate (isotropic) treatment of cell esds is used for estimating esds involving l.s. planes.
Refinement. Refinement was performed using all reflections. The weighted R-factor (wR) and goodness of fit (S) are based on F^2^. R-factor (gt) are based on F. The threshold expression of F^2^ > 2.0 sigma(F^2^) is used only for calculating R-factor (gt).

### Fractional atomic coordinates and isotropic or equivalent isotropic displacement parameters (Å^2^)

**Table:**

	*x*	*y*	*z*	*U*_iso_*/*U*_eq_	Occ. (<1)
Br1	0.32389 (2)	−0.22866 (2)	−0.02368 (2)	0.04191 (8)	
N2	0.10884 (14)	−0.03518 (6)	0.24791 (14)	0.0214 (3)	
N3	0.13607 (14)	0.05539 (6)	0.44000 (14)	0.0194 (3)	
C4	−0.00730 (16)	0.05269 (6)	0.37843 (15)	0.0178 (3)	
C5	−0.07237 (16)	0.09426 (6)	0.43609 (15)	0.0171 (3)	
C6	−0.21856 (16)	0.10061 (7)	0.38848 (16)	0.0190 (3)	
H6	−0.2609	0.1292	0.4243	0.023*	
C7	−0.30146 (16)	0.06474 (7)	0.28826 (16)	0.0202 (3)	
C8	−0.23344 (17)	0.02255 (7)	0.23600 (16)	0.0210 (3)	
H8	−0.2887	−0.0021	0.1703	0.025*	
C9	−0.08739 (16)	0.01562 (6)	0.27729 (16)	0.0187 (3)	
C10	0.16594 (16)	0.09673 (6)	0.54128 (16)	0.0183 (3)	
C11	0.03872 (15)	0.12215 (6)	0.54154 (15)	0.0169 (3)	
C12	0.03888 (16)	0.16442 (6)	0.63623 (15)	0.0172 (3)	
H12	−0.0456	0.1811	0.6359	0.021*	
C13	0.16444 (16)	0.18174 (6)	0.73110 (15)	0.0183 (3)	
C14	0.29004 (16)	0.15590 (7)	0.72658 (16)	0.0213 (3)	
H14	0.3748	0.1675	0.7894	0.026*	
C15	0.29330 (16)	0.11409 (7)	0.63310 (17)	0.0216 (3)	
H15	0.3781	0.0982	0.6319	0.026*	
C16	−0.46276 (17)	0.06969 (7)	0.23445 (18)	0.0249 (4)	
C17	−0.5292 (2)	0.01470 (9)	0.2628 (2)	0.0423 (5)	
H17A	−0.5002	0.0079	0.3597	0.051*	
H17B	−0.4991	−0.0163	0.2182	0.051*	
H17C	−0.6300	0.0179	0.2285	0.051*	
C18	−0.5096 (2)	0.07977 (9)	0.07942 (19)	0.0351 (4)	
H18A	−0.6106	0.0811	0.0447	0.042*	
H18B	−0.4758	0.0494	0.0359	0.042*	
H18C	−0.4716	0.1152	0.0606	0.042*	
C19	−0.51743 (19)	0.11897 (9)	0.2992 (2)	0.0386 (5)	
H19A	−0.4766	0.1538	0.2812	0.046*	
H19B	−0.4920	0.1132	0.3963	0.046*	
H19C	−0.6182	0.1210	0.2610	0.046*	
C20	0.16630 (17)	0.22683 (7)	0.83868 (17)	0.0226 (3)	
C21A	0.2398 (4)	0.20070 (14)	0.9822 (3)	0.0349 (8)	0.592 (3)
H21A	0.2411	0.2281	1.0517	0.042*	0.592 (3)
H21B	0.3347	0.1903	0.9893	0.042*	0.592 (3)
H21C	0.1890	0.1676	0.9943	0.042*	0.592 (3)
C22A	0.0240 (4)	0.24682 (17)	0.8322 (4)	0.0458 (11)	0.592 (3)
H22A	−0.0202	0.2644	0.7452	0.055*	0.592 (3)
H22B	0.0317	0.2739	0.9040	0.055*	0.592 (3)
H22C	−0.0318	0.2151	0.8434	0.055*	0.592 (3)
C23A	0.2585 (4)	0.27633 (13)	0.8214 (3)	0.0344 (8)	0.592 (3)
H23A	0.2170	0.2935	0.7333	0.041*	0.592 (3)
H23B	0.3509	0.2625	0.8284	0.041*	0.592 (3)
H23C	0.2655	0.3040	0.8917	0.041*	0.592 (3)
C21B	0.0816 (5)	0.2036 (2)	0.9295 (4)	0.0310 (11)	0.408 (3)
H21D	0.1277	0.1705	0.9775	0.037*	0.408 (3)
H21E	−0.0118	0.1937	0.8731	0.037*	0.408 (3)
H21F	0.0762	0.2321	0.9943	0.037*	0.408 (3)
C22B	0.0843 (5)	0.28167 (19)	0.7672 (5)	0.0324 (11)	0.408 (3)
H22D	0.0760	0.3078	0.8359	0.039*	0.408 (3)
H22E	−0.0080	0.2711	0.7094	0.039*	0.408 (3)
H22F	0.1351	0.2994	0.7130	0.039*	0.408 (3)
C23B	0.3089 (4)	0.2460 (2)	0.9280 (5)	0.0346 (12)	0.408 (3)
H23D	0.3601	0.2140	0.9772	0.041*	0.408 (3)
H23E	0.2979	0.2739	0.9917	0.041*	0.408 (3)
H23F	0.3599	0.2622	0.8721	0.041*	0.408 (3)
C24	−0.02473 (16)	−0.02866 (7)	0.21740 (16)	0.0197 (3)	
H24	−0.0838	−0.0533	0.1544	0.024*	
C25	0.15800 (17)	−0.07912 (7)	0.18052 (16)	0.0207 (3)	
C26	0.26499 (17)	−0.11434 (7)	0.25860 (18)	0.0235 (3)	
H26	0.3037	−0.1079	0.3521	0.028*	
C27	0.31439 (17)	−0.15892 (7)	0.19842 (19)	0.0263 (4)	
H27	0.3834	−0.1833	0.2514	0.032*	
C28	0.25953 (18)	−0.16661 (7)	0.05853 (19)	0.0263 (4)	
C29	0.15945 (19)	−0.13042 (8)	−0.02231 (19)	0.0288 (4)	
H29	0.1269	−0.1351	−0.1168	0.035*	
C30	0.10778 (18)	−0.08681 (7)	0.03954 (17)	0.0261 (4)	
H30	0.0389	−0.0625	−0.0140	0.031*	
H3	0.187 (2)	0.0341 (9)	0.426 (2)	0.027 (5)*	

### Atomic displacement parameters (Å^2^)

**Table:**

	*U*^11^	*U*^22^	*U*^33^	*U*^12^	*U*^13^	*U*^23^
Br1	0.03992 (12)	0.03025 (11)	0.05975 (15)	0.00525 (8)	0.02168 (10)	−0.01567 (9)
N2	0.0257 (7)	0.0183 (6)	0.0222 (7)	−0.0004 (5)	0.0103 (6)	−0.0001 (5)
N3	0.0172 (6)	0.0193 (7)	0.0222 (7)	0.0024 (5)	0.0069 (5)	−0.0017 (5)
C4	0.0192 (7)	0.0168 (7)	0.0181 (7)	0.0000 (6)	0.0066 (6)	0.0036 (6)
C5	0.0208 (7)	0.0157 (7)	0.0154 (7)	−0.0007 (6)	0.0065 (6)	0.0015 (6)
C6	0.0196 (7)	0.0182 (7)	0.0194 (8)	0.0025 (6)	0.0065 (6)	0.0007 (6)
C7	0.0185 (8)	0.0211 (8)	0.0198 (8)	0.0005 (6)	0.0045 (6)	0.0009 (6)
C8	0.0229 (8)	0.0183 (7)	0.0202 (8)	−0.0021 (6)	0.0045 (6)	−0.0017 (6)
C9	0.0225 (8)	0.0161 (7)	0.0188 (7)	0.0000 (6)	0.0082 (6)	0.0016 (6)
C10	0.0214 (8)	0.0160 (7)	0.0186 (7)	0.0009 (6)	0.0078 (6)	0.0021 (6)
C11	0.0166 (7)	0.0165 (7)	0.0175 (7)	−0.0006 (6)	0.0053 (6)	0.0035 (6)
C12	0.0163 (7)	0.0166 (7)	0.0193 (7)	0.0007 (6)	0.0063 (6)	0.0020 (6)
C13	0.0200 (7)	0.0178 (7)	0.0178 (7)	−0.0018 (6)	0.0068 (6)	0.0018 (6)
C14	0.0164 (7)	0.0246 (8)	0.0207 (8)	−0.0023 (6)	0.0025 (6)	0.0006 (6)
C15	0.0159 (7)	0.0234 (8)	0.0258 (8)	0.0029 (6)	0.0067 (6)	0.0020 (7)
C16	0.0175 (8)	0.0246 (8)	0.0291 (9)	−0.0008 (6)	0.0023 (7)	−0.0044 (7)
C17	0.0228 (9)	0.0388 (11)	0.0652 (14)	−0.0008 (8)	0.0135 (9)	0.0078 (10)
C18	0.0256 (9)	0.0400 (11)	0.0324 (10)	0.0030 (8)	−0.0013 (8)	−0.0039 (8)
C19	0.0195 (8)	0.0479 (12)	0.0426 (11)	0.0070 (8)	0.0017 (8)	−0.0161 (9)
C20	0.0229 (8)	0.0219 (8)	0.0220 (8)	−0.0047 (6)	0.0058 (6)	−0.0037 (6)
C21A	0.049 (2)	0.0336 (17)	0.0241 (16)	−0.0039 (14)	0.0149 (14)	−0.0042 (13)
C22A	0.0303 (17)	0.047 (2)	0.054 (2)	0.0007 (15)	0.0055 (16)	−0.0364 (19)
C23A	0.0448 (19)	0.0241 (15)	0.0292 (17)	−0.0123 (13)	0.0045 (14)	−0.0049 (13)
C21B	0.039 (3)	0.035 (2)	0.021 (2)	−0.012 (2)	0.0112 (19)	−0.0070 (18)
C22B	0.045 (3)	0.026 (2)	0.026 (2)	0.0088 (19)	0.012 (2)	−0.0038 (18)
C23B	0.021 (2)	0.038 (3)	0.043 (3)	−0.0064 (18)	0.006 (2)	−0.016 (2)
C24	0.0243 (8)	0.0169 (7)	0.0179 (7)	−0.0011 (6)	0.0066 (6)	0.0000 (6)
C25	0.0229 (8)	0.0171 (7)	0.0248 (8)	−0.0020 (6)	0.0114 (7)	−0.0019 (6)
C26	0.0199 (8)	0.0268 (8)	0.0244 (8)	−0.0016 (6)	0.0080 (7)	−0.0007 (7)
C27	0.0202 (8)	0.0238 (8)	0.0368 (10)	0.0024 (6)	0.0117 (7)	0.0028 (7)
C28	0.0258 (8)	0.0194 (8)	0.0389 (10)	−0.0004 (6)	0.0175 (8)	−0.0067 (7)
C29	0.0342 (9)	0.0286 (9)	0.0244 (9)	0.0021 (7)	0.0104 (7)	−0.0050 (7)
C30	0.0306 (9)	0.0232 (8)	0.0244 (9)	0.0054 (7)	0.0084 (7)	0.0016 (7)

### Geometric parameters (Å, º)

**Table:**

Br1—C28	1.8992 (16)	C19—H19B	0.9600
N2—C24	1.281 (2)	C19—H19C	0.9600
N2—C25	1.417 (2)	C20—C22A	1.479 (4)
N3—C4	1.374 (2)	C20—C23B	1.502 (4)
N3—C10	1.388 (2)	C20—C23A	1.530 (3)
N3—H3	0.76 (2)	C20—C21B	1.545 (5)
C4—C9	1.401 (2)	C20—C21A	1.555 (4)
C4—C5	1.406 (2)	C20—C22B	1.581 (5)
C5—C6	1.395 (2)	C21A—H21A	0.9600
C5—C11	1.444 (2)	C21A—H21B	0.9600
C6—C7	1.388 (2)	C21A—H21C	0.9600
C6—H6	0.9300	C22A—H22A	0.9600
C7—C8	1.403 (2)	C22A—H22B	0.9600
C7—C16	1.535 (2)	C22A—H22C	0.9600
C8—C9	1.396 (2)	C23A—H23A	0.9600
C8—H8	0.9300	C23A—H23B	0.9600
C9—C24	1.450 (2)	C23A—H23C	0.9600
C10—C15	1.387 (2)	C21B—H21D	0.9600
C10—C11	1.406 (2)	C21B—H21E	0.9600
C11—C12	1.393 (2)	C21B—H21F	0.9600
C12—C13	1.387 (2)	C22B—H22D	0.9600
C12—H12	0.9300	C22B—H22E	0.9600
C13—C14	1.409 (2)	C22B—H22F	0.9600
C13—C20	1.530 (2)	C23B—H23D	0.9600
C14—C15	1.384 (2)	C23B—H23E	0.9600
C14—H14	0.9300	C23B—H23F	0.9600
C15—H15	0.9300	C24—H24	0.9300
C16—C19	1.523 (2)	C25—C30	1.390 (2)
C16—C17	1.525 (3)	C25—C26	1.392 (2)
C16—C18	1.533 (3)	C26—C27	1.386 (2)
C17—H17A	0.9600	C26—H26	0.9300
C17—H17B	0.9600	C27—C28	1.382 (3)
C17—H17C	0.9600	C27—H27	0.9300
C18—H18A	0.9600	C28—C29	1.377 (3)
C18—H18B	0.9600	C29—C30	1.390 (2)
C18—H18C	0.9600	C29—H29	0.9300
C19—H19A	0.9600	C30—H30	0.9300
			
C24—N2—C25	117.55 (14)	C13—C20—C23A	108.37 (17)
C4—N3—C10	108.99 (13)	C23B—C20—C21B	109.3 (3)
C4—N3—H3	123.0 (15)	C13—C20—C21B	107.9 (2)
C10—N3—H3	127.4 (15)	C22A—C20—C21A	109.2 (2)
N3—C4—C9	129.86 (14)	C13—C20—C21A	107.92 (17)
N3—C4—C5	109.09 (13)	C23A—C20—C21A	106.8 (2)
C9—C4—C5	121.03 (14)	C23B—C20—C22B	107.0 (3)
C6—C5—C4	119.92 (14)	C13—C20—C22B	110.1 (2)
C6—C5—C11	133.48 (14)	C21B—C20—C22B	105.5 (3)
C4—C5—C11	106.59 (13)	C20—C21A—H21A	109.5
C7—C6—C5	120.68 (14)	C20—C21A—H21B	109.5
C7—C6—H6	119.7	H21A—C21A—H21B	109.5
C5—C6—H6	119.7	C20—C21A—H21C	109.5
C6—C7—C8	117.89 (14)	H21A—C21A—H21C	109.5
C6—C7—C16	122.38 (14)	H21B—C21A—H21C	109.5
C8—C7—C16	119.73 (14)	C20—C22A—H22A	109.5
C9—C8—C7	123.55 (15)	C20—C22A—H22B	109.5
C9—C8—H8	118.2	H22A—C22A—H22B	109.5
C7—C8—H8	118.2	C20—C22A—H22C	109.5
C8—C9—C4	116.87 (14)	H22A—C22A—H22C	109.5
C8—C9—C24	120.32 (14)	H22B—C22A—H22C	109.5
C4—C9—C24	122.80 (14)	C20—C23A—H23A	109.5
C15—C10—N3	130.74 (14)	C20—C23A—H23B	109.5
C15—C10—C11	120.66 (14)	H23A—C23A—H23B	109.5
N3—C10—C11	108.59 (13)	C20—C23A—H23C	109.5
C12—C11—C10	120.24 (14)	H23A—C23A—H23C	109.5
C12—C11—C5	133.04 (14)	H23B—C23A—H23C	109.5
C10—C11—C5	106.69 (13)	C20—C21B—H21D	109.5
C13—C12—C11	120.29 (14)	C20—C21B—H21E	109.5
C13—C12—H12	119.9	H21D—C21B—H21E	109.5
C11—C12—H12	119.9	C20—C21B—H21F	109.5
C12—C13—C14	117.91 (14)	H21D—C21B—H21F	109.5
C12—C13—C20	121.06 (14)	H21E—C21B—H21F	109.5
C14—C13—C20	121.03 (14)	C20—C22B—H22D	109.5
C15—C14—C13	123.11 (14)	C20—C22B—H22E	109.5
C15—C14—H14	118.4	H22D—C22B—H22E	109.5
C13—C14—H14	118.4	C20—C22B—H22F	109.5
C14—C15—C10	117.77 (14)	H22D—C22B—H22F	109.5
C14—C15—H15	121.1	H22E—C22B—H22F	109.5
C10—C15—H15	121.1	C20—C23B—H23D	109.5
C19—C16—C17	109.00 (16)	C20—C23B—H23E	109.5
C19—C16—C18	107.66 (15)	H23D—C23B—H23E	109.5
C17—C16—C18	108.79 (15)	C20—C23B—H23F	109.5
C19—C16—C7	112.37 (14)	H23D—C23B—H23F	109.5
C17—C16—C7	109.72 (14)	H23E—C23B—H23F	109.5
C18—C16—C7	109.22 (14)	N2—C24—C9	122.55 (15)
C16—C17—H17A	109.5	N2—C24—H24	118.7
C16—C17—H17B	109.5	C9—C24—H24	118.7
H17A—C17—H17B	109.5	C30—C25—C26	119.00 (15)
C16—C17—H17C	109.5	C30—C25—N2	122.63 (15)
H17A—C17—H17C	109.5	C26—C25—N2	118.32 (14)
H17B—C17—H17C	109.5	C27—C26—C25	120.68 (16)
C16—C18—H18A	109.5	C27—C26—H26	119.7
C16—C18—H18B	109.5	C25—C26—H26	119.7
H18A—C18—H18B	109.5	C28—C27—C26	118.97 (16)
C16—C18—H18C	109.5	C28—C27—H27	120.5
H18A—C18—H18C	109.5	C26—C27—H27	120.5
H18B—C18—H18C	109.5	C29—C28—C27	121.56 (15)
C16—C19—H19A	109.5	C29—C28—Br1	119.37 (14)
C16—C19—H19B	109.5	C27—C28—Br1	119.07 (13)
H19A—C19—H19B	109.5	C28—C29—C30	118.97 (16)
C16—C19—H19C	109.5	C28—C29—H29	120.5
H19A—C19—H19C	109.5	C30—C29—H29	120.5
H19B—C19—H19C	109.5	C25—C30—C29	120.66 (16)
C22A—C20—C13	113.32 (17)	C25—C30—H30	119.7
C23B—C20—C13	116.4 (2)	C29—C30—H30	119.7
C22A—C20—C23A	111.0 (2)		
			
C10—N3—C4—C9	−175.87 (15)	N3—C10—C15—C14	−178.10 (16)
C10—N3—C4—C5	2.32 (17)	C11—C10—C15—C14	1.7 (2)
N3—C4—C5—C6	178.89 (13)	C6—C7—C16—C19	2.2 (2)
C9—C4—C5—C6	−2.7 (2)	C8—C7—C16—C19	−178.06 (16)
N3—C4—C5—C11	−1.96 (16)	C6—C7—C16—C17	−119.19 (18)
C9—C4—C5—C11	176.41 (14)	C8—C7—C16—C17	60.5 (2)
C4—C5—C6—C7	2.6 (2)	C6—C7—C16—C18	121.64 (17)
C11—C5—C6—C7	−176.26 (16)	C8—C7—C16—C18	−58.7 (2)
C5—C6—C7—C8	−0.6 (2)	C12—C13—C20—C22A	2.2 (3)
C5—C6—C7—C16	179.10 (14)	C14—C13—C20—C22A	−176.9 (2)
C6—C7—C8—C9	−1.4 (2)	C12—C13—C20—C23B	−174.8 (3)
C16—C7—C8—C9	178.89 (15)	C14—C13—C20—C23B	6.2 (3)
C7—C8—C9—C4	1.3 (2)	C12—C13—C20—C23A	−121.4 (2)
C7—C8—C9—C24	−179.34 (15)	C14—C13—C20—C23A	59.5 (2)
N3—C4—C9—C8	178.81 (15)	C12—C13—C20—C21B	61.9 (3)
C5—C4—C9—C8	0.8 (2)	C14—C13—C20—C21B	−117.1 (2)
N3—C4—C9—C24	−0.6 (3)	C12—C13—C20—C21A	123.2 (2)
C5—C4—C9—C24	−178.55 (14)	C14—C13—C20—C21A	−55.9 (2)
C4—N3—C10—C15	178.11 (16)	C12—C13—C20—C22B	−52.8 (3)
C4—N3—C10—C11	−1.74 (17)	C14—C13—C20—C22B	128.1 (2)
C15—C10—C11—C12	−1.3 (2)	C25—N2—C24—C9	−178.58 (14)
N3—C10—C11—C12	178.55 (13)	C8—C9—C24—N2	177.08 (15)
C15—C10—C11—C5	−179.37 (14)	C4—C9—C24—N2	−3.6 (2)
N3—C10—C11—C5	0.50 (17)	C24—N2—C25—C30	48.4 (2)
C6—C5—C11—C12	2.2 (3)	C24—N2—C25—C26	−134.37 (16)
C4—C5—C11—C12	−176.82 (16)	C30—C25—C26—C27	−4.4 (2)
C6—C5—C11—C10	179.87 (16)	N2—C25—C26—C27	178.28 (14)
C4—C5—C11—C10	0.88 (16)	C25—C26—C27—C28	2.5 (2)
C10—C11—C12—C13	−0.1 (2)	C26—C27—C28—C29	1.3 (3)
C5—C11—C12—C13	177.38 (15)	C26—C27—C28—Br1	−178.58 (12)
C11—C12—C13—C14	0.9 (2)	C27—C28—C29—C30	−3.1 (3)
C11—C12—C13—C20	−178.14 (14)	Br1—C28—C29—C30	176.80 (13)
C12—C13—C14—C15	−0.5 (2)	C26—C25—C30—C29	2.5 (3)
C20—C13—C14—C15	178.59 (15)	N2—C25—C30—C29	179.78 (15)
C13—C14—C15—C10	−0.8 (2)	C28—C29—C30—C25	1.1 (3)

### Hydrogen-bond geometry (Å, º)

*Cg*1, *Cg*2 and *Cg*3 are the centroids of the C25–C30, C4–C9 and N3/C4/C5/C11/C10 rings, respectively.

**Table:**

*D*—H···*A*	*D*—H	H···*A*	*D*···*A*	*D*—H···*A*
N3—H3···N2	0.76 (2)	2.39 (2)	2.862 (2)	121.3 (16)
C22*A*—H22*C*···*Cg*1^i^	0.96	2.92	3.878 (4)	177
C29—H29···*Cg*2^ii^	0.93	2.95	3.613 (2)	129
C21*B*—H21*E*···*Cg*1^i^	0.96	2.62	3.391 (5)	138
C22*B*—H22*D*···*Cg*3^iii^	0.96	2.92	3.839 (5)	159

Symmetry codes: (i) −*x*, −*y*, −*z*+1; (ii) −*x*, −*y*, −*z*; (iii) *x*, −*y*−1/2, *z*−1/2.

## References

[bb1] Altomare, A., Cascarano, G., Giacovazzo, C. & Guagliardi, A. (1993). *J. Appl. Cryst.* **26**, 343–350.

[bb2] Gibson, V. C., Spitzmesser, S. K., White, A. J. P. & Williams, D. J. (2003). *Dalton Trans.* pp. 2718.

[bb3] Groom, C. R., Bruno, I. J., Lightfoot, M. P. & Ward, S. C. (2016). *Acta Cryst.* B**72**, 171–179. 10.1107/S2052520616003954 PMC4822653 27048719

[bb4] Krucaite, G. & Grigalevicius, S. (2019). *Synth. Met.* **247**, 90–108.

[bb5] Nolla-Saltiel, R., Geer, A. M., Lewis, W., Blake, A. J. & Kays, D. L. (2018). *Chem. Commun.* **54**, 1825–1828. 10.1039/c7cc08385h 29322123

[bb6] Obora, Y. (2018). *Tetrahedron Lett.* **59**, 167–172.

[bb7] Rigaku (2016). *CrystalStructure*. Rigaku Corporation, Tokyo, Japan.

[bb8] Rigaku OD (2018). *CrysAlis PRO*. Rigaku Corporation, Tokyo, Japan.

[bb9] Rubio, O. H., Fuentes de Arriba, L., Monleón, L. M., Sanz, F., Simón, L., Alcázar, V. & Morán, J. R. (2015). *Tetrahedron*, **71**, 1297–1303.

[bb10] Sheldrick, G. M. (2015). *Acta Cryst.* C**71**, 3–8.

[bb11] Spek, A. L. (2009). *Acta Cryst.* D**65**, 148–155. 10.1107/S090744490804362X PMC2631630 19171970

[bb12] Taneda, M., Shizu, K., Tanaka, H. & Adachi, C. (2015). *Chem. Commun.* **51**, 5028–5031. 10.1039/c5cc00511f 25705974

[bb13] Wiosna-Sałyga, G., Dobkowski, J., Mudadu, M. S., Sazanovich, I., Thummel, R. P. & Waluk, J. (2006). *Chem. Phys. Lett.* **423**, 288–292.

[bb14] Zhao, F., Chen, Z., Fan, C., Liu, G. & Pu, S. (2019). *Dyes Pigments*, **164**, 390–397.

